# High resolution human leukocyte antigen (HLA) class I and class II allele typing in Mexican mestizo women with sporadic breast cancer: case-control study

**DOI:** 10.1186/1471-2407-9-48

**Published:** 2009-02-05

**Authors:** David Cantú de León, Delia Pérez-Montiel, Verónica Villavicencio, Alejandro García Carranca, Alejandro Mohar Betancourt, Victor Acuña-Alonzo, Alberto López-Tello, Gilberto Vargas-Alarcón, Rodrigo Barquera, Neng Yu, Edmond J Yunis, Julio Granados

**Affiliations:** 1 Department of Gynecologic Oncology, Instituto Nacional de Cancerología de México, Mexico City, Mexico; 2 Department of Pathology, Instituto Nacional de Cancerologia, México City, México; 3 Division of Surgery, Insituto Nacional de Cancerología, México City, México; 4 Laboratory of Virus and Cancer, Instituto de Investigaciones Biomédicas, Universidad Nacional Autónoma de México, México City, México; 5 Unidad de investigacion biomedica, Instituto Nacional de Cancerologia, México City, México; 6 Molecular Genetics Laboratory, ENAH, México City, México; 7 Immunology and Rehumatology Department, Instituto Nacional de Ciencias Médicas y Nutrición " Salvador Zubirán ", Mexico City, Mexico; 8 Department of Physiology. Instituto Nacional de Cardiología "Ignacio Chavez" Mexico City, Mexico; 9 American Red Cross Blood Services, New England Region, Dedham, MA, USA; 10 Department of Cancer Immunology and AIDS, Dana-Farber Cancer Institute, Boston, MA 02115, USA

## Abstract

**Background:**

The development of breast cancer is multifactorial. Hormonal, environmental factors and genetic predisposition, among others, could interact in the presentation of breast carcinoma. Human leukocyte antigen (HLA) alleles play an important role in immunity (cellular immunity) and may be important genetic traits. HLAAllele-specific interaction has not been well established. Recently, several studies had been conducted in order to do so, but the results are controversial and in some instances contradictory.

**Methods:**

We designed a case-control study to quantify the association of HLA class I and II genes and breast cancer. HLA typing was performed by high resolution sequence-specific oligotyping after DNA amplification (PCR-SSOP) of 100 breast cancer Mexican mestizo patients and 99 matched healthy controls.

**Results:**

HLA-A frequencies that we were able to observe that there was no difference between both groups from the statistical viewpoint. HLA-B*1501 was found three times more common in the case group (OR, 3.714; *p *= 0.031). HLA-Cw is not a marker neither for risk, nor protection for the disease, because we did not find significant statistical differences between the two groups. DRB1*1301, which is expressed in seven cases and in only one control, observing an risk increase of up to seven times and DRB1*1602, which behaves similarly in being present solely in the cases (OR, 16.701; 95% CI, 0.947 – 294.670). DQ*0301-allele expression, which is much more common in the control group and could be protective for the presentation of the disease (OR, 0.078; 95% CI, 0.027–0.223, *p *= 0.00001).

**Conclusion:**

Our results reveal the role of the MHC genes in the pathophysiology of breast cancer, suggesting that in the development of breast cancer exists a disorder of immune regulation. The triggering factor seems to be restricted to certain ethnic groups and certain geographical regions since the relevant MHC alleles are highly diverse. This is the first study in Mexican population where high resolutions HLA typing has been performed in order to try to establish an association with malignancy.

## Background

Breast cancer is a common neoplasm around the world with almost 1 million cases diagnosed every year, it is also considered the most frequent malignant neoplasm in developed countries, globally accounts for 18% of all female cancers [1]. In Mexico, this neoplasm occupies second place, preceded only by cancer of the cervix, which occupies 10.6% of all tumors and 16.4% of all tumors in women. It is considered that the combination of cervico-uterine cancer and breast cancer corresponds to 49% of all neoplasms in Mexican women [2].

Multiple factors are associated with an increase in breast cancer development, including age, family history, exposure to hormones (endo – as well as exogenous), diet, benign mammary disease, and environmental and genetic factors. The majority of these factors moderately increase the risk of developing cancer. It is estimated that at least 50 and up to 80% of women who develop breast cancer do not possess predisposing factors in addition to gender and age [3].

According to Rodríguez-Cuevas et al. [4], in Mexico from 1993 – 1995, 29,075 new cases of breast cancer were reported, of which 45.5% presented at the age of < 50 years; it is noteworthy that the most affected age group was that of 40 – 49 years, corresponding to 29.5% of all tumors. When a comparison was carried out with studies reported by other authors, it was found that in Mexico, this disease presented at least one decade prior to presentation in European countries or in the U.S. On conducting a comparative evaluation with other Latin American countries such as Venezuela [5], we found that the percentage of women < 50 years of age with a diagnosis of breast cancer is similar to that of Mexico. Thus, we concluded that Latin American women have the tendency to develop this type of neoplasm at an earlier age [4,5].

This observation is similar to that for Japanese women, in whom 46.5% of women with this disease were aged < 50 years [6]. It appears that environmental or dietary factors are not responsible for this behavior, because reports in the literature evaluating Hispanic patients residing in Los Angeles, California, or in the U.S. state of New Mexico show a percentage of presentation (38 – 39%) similar to that of ages of women living in Mexico or other Latin American countries [7]. According to this information, it is possible that there is (are) some factor(s) that make(s) women present this disease when no other risk factor is found.

One of the predisposing factors can be genes located within the major histocompatibility complex (MHC) region; the association between human leukocyte antigen (HLA) gene products with a disease does not necessarily reflect the direct involvement of these molecules in the disease process [8], and because many genes can be in linkage disequilibrium with other MHC genes, this possible association could be due solely to a closely associated gene.

The HLA system involvement in the development of cancer is poorly understood; nonetheless, it is widely recognized that MHC genetic variations lead to greater susceptibility to neoplasm development [9]. Neoplastic cells express a number of genes not expressed by their normal counterpart, and also some peptides of some proteic products of these HLA molecule-associated genes [10].

The role of oncogene – and tumor suppressor gene-acquired changes is widely recognized; similarly, there is growing evidence suggesting that the immune system plays a protector role in tumorigenesis [11,12]. In patients with cancer, HLA peptide complex-stimulated T-cell responses are not sufficiently effective for eliminating tumor cells. Loss of HLA expression or deregulation has been reported in a great variety of tumors, including breast cancer [13]; changes in the expression of these antigens have been associated with poor prognosis. Notwithstanding this, in tumoral tissue class I antigen expression is rarely lost in its entirety [14]. Such changes connote the possibility that this represents mechanisms by which neoplastic cells escape cell-mediated immunological surveillance – due to their being poor targets for cytotoxic T-cells – allowing for tumor dissemination and metastasis [15].

If immunological surveillance is important during tumorigenesis, certain individuals who inherit specific HLA class I alleles, which are highly polymorphic, such as DRB or DQB, can be more susceptible to developing tumors, or contrariwise, more resistant to the growth of these [13,14].

In breast cancer, the study of HLA is reduced; the greatest number of studies is conducted on HLA class I expression. These studies have shown that up to 80% of tumors exhibit partial or total loss of HLA class I antigens [16,17], while other tumors such as cervix, larynx, melanoma, colon, and pancreas demonstrate a loss of up to 40 – 50% [18-20]. Evaluation has arrived at the field of prognosis; for example, in the study of Gudmundsdottir et al. [21], the authors showed that a cohort of 187 patients with clinical stages I and II, mixed HLA class I expression exhibited an increase in the probability of late recurrence and a greater probability of death (odds ratio [OR] = 3.42; *p *= 0.014) due to the disease in patients with negative auxiliary lymph nodes in comparison with patients demonstrating total negativity or positivity, especially after 5 years.

The first evaluation of HLA class II and their alleles was carried out by Chaudhuri et al. [13] in a group of 173 patient with breast cancer and 215 Caucasian-origin controls, showing the presence of the DRB3*0201/*0202 allele in 55% of cases and in 40.9% of controls (*p *= 0.0072) as risk factor. At the same time, the authors concluded that DQB*03032 and DRB1*11 alleles represent resistance factors toward the disease. HLA polymorphisms appear to be responsible for the immune response variations in different individuals to different antigens and can contribute to susceptibility to the disease, specifically to non virus-related tumors, because breast cancer frequency in Mexico is high, thus considered a health problem, and the disease is present in any age group, with the characteristic of presenting at an earlier age than in other countries, and with the evidence that between 50 and 80% [1,3] of patients do not present the classical risk factors of the disease; therefore, it is necessary to investigate whether there is loss of control of the immune system regarding the tumor cell in this group of sick persons that allows neoplastic growth. At present, there are no reports of HLA system alleles in Mexican mestizo female population with breast cancer.

## Methods

### Subjects

We developed a case-control study at the National Institute of Cancerology (Instiuto Nacional de Cancerología de México, INCan) in Mexico City. A case was defined as a Mexican mestizo female patient with at least two previous generations born in Mexico, in whom breast cancer confirmed by histopathology has been diagnosed, who has been treated at the INCan Breast Tumor Service. A control was defined as a Mexican mestizo female patient who has at least two previous generations born in Mexico, from open population, without a family history of any type of cancer, with emphasis placed on breast, colon, ovary, and prostate cancer, without a history of autoimmune diseases, who has been submitted to breast and/or radiological exploration that discard pathology at this level according to patient age. We applied a clinical history oriented toward determination of personal and familial antecedents-of-interest; in the case of obtaining no response being or the response being positive, the patient was excluded from the study.

Determination of the absence of mammary pathology was performed according to patient age, with the following American Cancer Society (ACS) guidelines for detection of early breast cancer [22]: a) In women < 40 years of age, a clinical examination was conducted exclusively; in the case of requiring further evaluation, the patient was discarded as a control and excluded from the study, and b) in women aged > 40 years, we carried out a clinical examination as well as a mammographic study and breast ultrasound (US) to determine mammary pathology. In the case of obtaining an abnormal result or requiring further examination, the patient was discarded as a control.

The study was evaluated and approved by the Scientific and Ethical Committee of the Instituto Nacional de Cancerología de México, and all patients who were evaluated provided informed consent for radiographic studies, the taking of blood samples, and evaluation of genetic material. This study was performed in collaboration and with the technical and methodological support of the American Red Cross in Nedham Massachusetts, USA.

### HLA typing

Genomic DNA was obtained from peripheral blood leukocytes and extracted by standard techniques [23,24].

### Amplification of genomic DNA

HLA-DQA1 and – DQB1 typing were amplified by PCR and hybridized to sequence specific oligonucleotide probes. Primers used for HLA-DQ amplification included DQAAMP-A,-B, DQBAMP-A, and -B. These were synthesized in a DNA-SM automated synthesizer (Beckman, Palo Alto, CA, USA). These typing techniques were approved by the 12th International Histocompatibility Workshop.

### Dot blot hybridization

Five percent of the amplified DNA was denatured in 0.4 mol/L NaOH for 10 min, neutralized in 1 mol/L of ammonium acetate, and transferred to a Hybond-N membrane (Amersham, Bucks, UK). The filters were prehybridized at 42°C for 30 min in a solution containing 6× SSPE (30× SSPE: 4.5 mol/L NaCl, 0.3 mol/L NaH2PO4, 30 mmol/L EDTA, pH = 7.4), 5× Denhard solution (2% bovine serum albumin, 2% polyvinylpyrrolidone 40, 2% Ficoll 400), 0.1% Lauryl-sarcosine, and 0.02% SDS. Then, the oligonucleotide probes labeled with Digoxygenin dideoxy- Uridine-Triphosphate (Dig-11-ddUTP) were added and hybridized at 42°C for 3 h. The filters were washed twice in 2× SSPE, 0.1% SDS at room temperature for 10 min, once in TMAC solution [50 mmol/L Tris-HCl (pH = 8.0), 3 mol/L tetramethylammonium chloride, 2 mmol/L EDTA, 0.1% SDS] at room temperature for 10 min, and twice at 60°C for 10 min. Dots were revealed using the Dig Nucleic Acid Detection Kit (Boehringer Mannheim Biochemical, Mannheim, Germany).

### Statistical analysis

HLA-A, HLA-B, HLA-C, allele and haplotype frequencies were estimated using the Arlequin program version 2.000 [25]. Significance of two-locus linkage disequilibrium (LD) was determined using Popgene program version 1.31 [26]. Odds ratio (OR) was calculated as per Haldane modified Woolf's formula [27]. OR = [(a + 0.5) (d + 0.5)/(b + 0.5) (c + 0.5)] where, a and b are the number of patients and controls positive for a given allele respectively, while c and d represent the number of patients and controls negative for the allele, respectively. The corrected P value was calculated using Bonferroni's inequality method [28] as, P corrected = 1- (1-p)n, where n = number of comparisons.

Association between HLA haplotype and breast cancer was examined using statistical analysis from a 2 × 2 table according to the method described by Svejgaard and Ryder [29].

## Results

During the study period, we included 100 patients who fulfilled inclusion and exclusion criteria with a confirmed diagnosis of breast cancer. Similarly, we obtained 99 samples of healthy control subjects.

Age of patients with breast cancer ranged from 27 – 82 years (average age, 50.4 ± 12.8 years); distribution was normal. Seventy two cases did not present a familiar history of breast cancer, and in 28 cases, there was at least one first-degree family member with this neoplasm type; average age at menarche was 12.8 years. Seventy one percent of women used no family planning method, while use of oral hormones or another hormonal-therapy type was present only in 29 patients; the remainder of patients utilized some other family planning method. History of smoking as a risk factor was present in only 16% of patients.

Locally advanced and advanced clinical stages were the most frequent (64%) stages in comparison with early stages. It is noteworthy that in 15% of cases, it was not possible to determine the clinical stage because the patients had been care for previously at another hospital. It was possible to determine tumor size in 89 cases, with an average of 5.2 cm (standard deviation [SD] ± 3.49; range, 1 – 17 cm); in addition, it was possible to determine the distribution of clinical lymph node status in 94 patients, the most frequent lymph node status being N1 with 46 cases, and the second most frequent, N0 with 23 patients, according to the Tumor-Node-Metastasis (TNM) lymph node staging description.

As expected due to neoplasm frequency, distribution by histological type obtained 94 cases of infiltrating ductal carcinoma and only six cases of infiltrating lobular carcinoma. Concerning differentiation degree, we found poorly differentiated carcinoma in 56% of cases, while moderately and well differentiated presented in 38 and 6% of cases, respectively. Distribution of differentiation degree with respect to the Scarff-Bloom-Richardson Index exhibited the presence of high-grade tumors in 67% of patients; the hormonal receptors of these tumors were distributed as follows: Positive estrogenic receptors in 54 cases; negative estrogenic receptors in 45 cases; positive pregestational receptors in 29 cases, and negative pregestational receptors in 70 cases. In one case, it was not possible to conduct hormonal receptor determination. Patient clinical characteristics were shown, as well as those of the neoplasms in Table 1.

**Table 1:** Patient Clinical Characteristics and Neoplasm Characteristics

		n
Age	50.4 +/- 12.8 (Mean +/- SD)	
		
Menarche	12.8 (Median)	
		
Family history	Positive	28
	Negative	72
		
Family planning	Positive for hormonal	29
	Negative	71
		
Tobbaco	Positive	16
		
Clinical stage	I	8
	IIa	13
	IIb	23
	IIIa	19
	IIIb	13
	IV	9
	No classified	15
		
Tumor size	5.2 +/- 3.49 (Mean +/- SD)	
		
Nodal status	N0	26
	N1	46
	N2	21
	N3	1
	Missing	6
		
Histology	Ductal	94
	Lobular	6
		
Grade	Well differentiated	6
	Moderately	38
	Poorly	56
		
Hormone receptor status	Estrogen positive	54
	Estrogen negative	45
	Progesterone positive	29
	Progesterone negative	70

At the moment of performing the present study, 61 patients were found without evidence of disease, while 39 cases presented disease recurrence (data not shown, in that this was not the objective of the present work).

Table 2 shows the different HLA classes I and II alleles studied in the group of cases, as well as their genetic frequencies. Table 3 depicts the different HLA classes I and II alleles studied in the control group of patients, as well as the genetic frequencies of these.

**Table 2:** Frequencies (g.f) of HLA-A,-B, Cw, DRB1 and – DQB1 in Cases.

-A	n	g.f	-B	n	g.f	-Cw	n	g.f	-DRB1	n	g.f	-DQB1	n	ggg.f g.f
				
0201	39	0.224	1501	13	0.074	0401	34	0.195	0802	27	0.155	0300	54	0.310
2402	25	0.143	3501	13	0.074	0702	29	0.167	0407	22	0.126	0302	30	0.172
0206	20	0.114	4002	11	0.063	0102	16	0.092	1406	14	0.080	0402	29	0.167
3101	16	0.091	5101	9	0.051	0701	13	0.075	0404	12	0.069	0200	18	0.103
6801	8	0.045	5201	9	0.051	0602	11	0.063	0301	12	0.069	0501	10	0.057
1101	8	0.045	3905	9	0.051	0303	9	0.052	0701	11	0.063	0602	6	0.034
6803	7	0.040	3512	8	0.046	0304	8	0.046	1301	7	0.040	0603	5	0.029
0101	7	0.040	0801	8	0.046	1203	7	0.040	1602	7	0.040	0601	5	0.029
0301	5	0.028	3517	7	0.040	1502	7	0.040	1501	6	0.034	0600	4	0.023
2601	5	0.028	3906	6	0.034	0801	6	0.034	0102	6	0.034	0303	4	0.023
3201	5	0.028	4801	6	0.034	0305	6	0.034	1402	5	0.029	0202	2	0.011
2902	5	0.028	0702	5	0.029	1509	5	0.029	0403	4	0.023	0503	2	0.011
3001	4	0.023	3801	5	0.029	1202	4	0.023	0410	4	0.023	0502	1	0.006
6802	3	0.017	4403	4	0.023	0802	4	0.023	1502	4	0.023	0201	1	0.006
2425	2	0.011	5001	4	0.023	1601	4	0.023	0402	3	0.017	0604	1	0.006
2501	2	0.011	1402	4	0.023	0202	3	0.017	1302	3	0.017			
3301	2	0.011	3514	4	0.023	1604	2	0.011	1101	3	0.017			
3010	1	0.005	4402	3	0.017	0803	2	0.011	0411	3	0.017			
0302	1	0.005	1530	3	0.017	0509	1	0.005	0401	2	0.011			
2301	1	0.005	1302	3	0.017	1801	1	0.005	0101	2	0.011			
6805	1	0.005	3508	3	0.017	1701	1	0.005	1104	2	0.011			
2201	1	0.005	4006	2	0.011	0501	1	0.005	1305	2	0.011			
7401	1	0.005	3908	2	0.011				0405	2	0.011			
3131	1	0.005	1515	2	0.011				1503	1	0.005			
6901	1	0.005	4101	2	0.011				1601	1	0.005			
3002	1	0.005	1801	2	0.011				1448	1	0.005			
0205	1	0.005	4501	2	0.011				1404	1	0.005			
2403	1	0.005	3503	2	0.011				1202	1	0.005			
			2705	2	0.011				0302	1	0.005			
			3905	2	0.011				0103	1	0.005			
			4008	1	0.005				1001	1	0.005			
			other	18	0.114				1401	1	0.005			

N = 174.

**Table 3:** Frequencies (g.f) of HLA-A,-B,-Cw,-DRB1 and – DQB1 in controls.

-A	n	g.f	-B	n	g.f	-Cw	n	g.f	-DRB1	n	g.f	-DBQ1	n	g.f
				
0201	41	0.220	3905	19	0.102	0702	40	0.215	0407	33	0.177	0302	51	0.274
2402	31	0.166	3512	14	0.075	0401	36	0.194	0802	25	0.134	0301	43	0.231
6801	19	0.102	4002	13	0.069	0304	16	0.086	0404	15	0.081	0402	28	0.151
3101	13	0.069	5101	11	0.059	0102	15	0.081	1406	15	0.081	Dqbx	21	0.113
AX	11	0.059	3501	11	0.059	Cwx	13	0.070	Drx	13	0.070	0501	12	0.065
0206	10	0.053	3906	10	0.053	0701	9	0.048	0701	12	0.065	0202	10	0.054
6803	8	0.03	BX	10	0.053	0602	8	0.043	1602	11	0.059	0201	6	0.032
3002	6	0.032	3514	6	0.032	0801	7	0.038	1501	7	0.038	0602	5	0.027
0301	6	0.032	4005	6	0.032	0802	7	0.038	1104	7	0.038	0603	3	0.016
3301	5	0.026	0702	6	0.032	0303	5	0.027	0301	6	0.032	0502	2	0.011
1101	4	0.021	4801	5	0.026	0501	4	0.022	0102	6	0.032	0303	2	0.011
0101	4	0.021	1402	5	0.026	1502	4	0.022	1402	5	0.027	0604	1	0.005
6802	4	0.021	5201	4	0.021	0305	3	0.016	0403	4	0.022	0601	1	0.005
2301	3	0.016	3543	4	0.021	1203	3	0.016	0101	3	0.016	0304	1	0.005
2601	3	0.016	0801	4	0.021	0202	3	0.016	0401	3	0.016			
2902	3	0.016	1501	4	0.021	0306	3	0.016	1001	3	0.016			
3201	3	0.016	3517	3	0.016	1601	2	0.010	0804	2	0.010			
6805	2	0.010	1515	3	0.016	1402	2	0.010	0411	2	0.010			
3001	2	0.010	1801	3	0.016	1509	2	0.010	0801	2	0.010			
0204	1	0.005	3902	3	0.016	0704	2	0.010	1407	1	0.005			
0224	1	0.005	3508	2	0.010	0401	1	0.005	1201	1	0.005			
0205	1	0.005	4901	2	0.010				1302	1	0.005			
6601	1	0.005	1401	2	0.010				1304	1	0.005			
0102	1	0.005	1516	2	0.010				1502	1	0.005			
2425	1	0.005	5301	2	0.010				0405	1	0.005			
2301/05	1	0.005	3701	2	0.010				1102	1	0.005			
			4402	2	0.010				0809	1	0.005			
2402/25	1	0.005	4501	2	0.010				1305	1	0.005			
			1302	2	0.010				1301	1	0.005			
			1517	2	0.010									
			3502	2	0.010									
			Other	20	0.107									

N = 186.

In Table 4, we found alleles with the highest genetic HLA-A frequencies that were detected; we were able to observe that there was no difference between both groups from the statistical viewpoint, although we noted a tendency for risk in one of these (*0206), as well as one for protection in the other (*6801), after correction for multiple comparisons for the number of alleles of HLA-A locus (n = 11), the risk was not significant (Pc = 0.45). It is worthwhile mentioning that the following four alleles were the most frequent in both groups: HLA-A*0201; -*2402; -*0206, and -*3101. In addition, also depicted in this Table are high-resolution HLA-B typifications with greatest genetic frequency compared – if only one exhibited a statistically significant difference for the risk factor, on finding this with a three times greater frequency in the case group in comparison with the control group HLA-B*1501 (OR, 3.714; *p *= 0.031). After correction for multiple comparisons for the number of alleles of HLA-B locus (n = 17), the risk was not significant (Pc = 0.30).

**Table 4:** Risk assessment among different loci of HLA class I

	CASES	N = 174	CONTROLS	N = 186			
Locus	n	g.f	n	g.f	P	OR	C195%
			
HLA-A							
0201	39	0.224	41	0.220	0.96	1.022	0.621–1.68
2402	25	0.143	31	0.166	0.648	0.839	0.473–1.48
**0206**	**20**	**0.114**	**10**	**0.053**	**0.056**	**2.286**	1.038–5.033*
3101	16	0.091	13	0.069	0.565	1.348	0.628–2.890
6801	8	0.045	19	0.102	0.068	0.424	0.180–0.995
							
HAL-B							
**1501**	**13**	**0.075**	**4**	**0.021**	**0.031**	**3.714**	**1.187–11.619+**
3501	13	0.075	11	0.059	0.704	1.285	0.560–2.949
4002	11	0.063	13	0.069	0.966	0.898	0.391–2.062
5101	9	0.052	11	0.059	0.939	0.868	0.351–2.148
5201	9	0.052	4	0.021	0.21	2.482	0.750–8.211
							
HLA-Cw							
0401	34	0.195	36	0.194	0.929	1.012	0.6–1.706
0702	29	0.167	40	0.215	0.302	0.73	0.430–1.241
0102	16	0.092	15	0.081	0.846	1.154	0.553–2.412
0701	13	0.075	9	0.048	0.411	1.588	0.661–3.814
0602	11	0.063	8	0.043	0.535	1.502	0.589–3.825

* Pc = 0.45. +Pc = 0.30.

HLA-Cw is a scarcely studied gene in this neoplasm type; we are able to say that at least in this group of women obtained from an ethnically similar population, HLA-Cw is not a marker for, nor a risk for, nor protection for the disease, because we did not find differences between the two groups.

In Table 5, we can observe HLA-DR distribution, in which we are able to identify two alleles that on being expressed comprise an associated risk factor for presenting the disease, such as DRB1*1301, which is expressed in seven cases and in only one control, observing an risk increase of up to seven times; notwithstanding this, it is important to mention that the confidence interval (CI) is very broad, which can be a reflection of its low genetic frequency (genetic frequency [g.f.] = 0.040) and DRB1*1602, which behaves similarly in being present solely in the cases (in seven of these) (OR, 16.701; 95% CI, 0.947 – 294.670), after correction for multiple comparisons for the number of alleles of HLA-DRB1 locus (n = 11), the risk was not significant (Pc = 0.24). Regarding HLA- DQ, we found two alleles of this gene associated with the disease, such as DQ*0302 with a g.f. of 0.454 in the group of cases, and a g.f. of 0.274 in the control group (OR, 2.201;95% CI,1.419–3.415), after correction for multiple comparisons for the number of alleles of HLA-DQ locus (n = 8), the risk was statistically significant (Pc = 0.0007). However, the allele commanding the majority of attention is DQ*0301-allele expression, which is much more common in the control group (g.f., of 0.231) being a protector presentation of the disease. This relationship is sustained after corrections for multiple comparisons (Pc = 0.00008) for HLA-DQB1 (n = 8).

**Table 5:** Risk assessment among different loci of HLA class II

	CASES	N = 174	CONTROLS	N = 186			
Locus	n	g.f	n	g.f	P	OR	C195%
		
HLA-DRB1							
0802	27	0.155	25	0.134	0.682	1.183	0.657–2.130
0407	22	0.126	33	0.177	0.231	0.671	0.374–1.204
1406	14	0.080	15	0.081	0.851	0.998	0.467–2.132
1301	7	0.040	1	0.005	0.06	7.754	0.944–63.689
**1602**	**7**	**0.040**	**0**	**0**	**0.025**	**16.701**	0.947–294.670*
							
HLA-DQB1							
**0302**	**79**	**0.454**	**51**	**0.274**	**0.0001**	**2.201**	1.419–3.415**
0402	29	0.167	28	0.151	0.784	1.129	0.641–1988
0202	18	0.103	10	0.054	0.118	2.031	0.910–4.531
**0301**	**4**	**0.022**	**43**	**0.231**	**0.00001**	**0.078**	0.027–0.223***
0201	2	0.011	0	0	0.524	5.406	0.258–113.402
0303	1	0.005	2	0.011	0.954	0.532	0.048–5.918

* Pc = 0.24. **Pc = 0.0007 ***Pc = 00008

Haplotypes were deduced both the results are highly heterogenic (data not show) therefore not conclusions could be drawn or associations performed.

## Discussion

The origin of malignant neoplasms is multifactorial [1]; nevertheless, there are certain factors that can increase not only the risk for appearance of the disease, but even more so that the tumor would continue to grow and would produce distal disease or metastasis. Thus, if immunological surveillance is an important mechanism in the tumor genesis process, certain individuals who inherit specific HLA class II alleles can be resistant or more susceptible to tumor presentation [13]. The results of different works show few reproducible results because there are important differences in the expression of the different HLAs, depending on the geographical area to which reference is made [34]. This is due to that the frequency of presentation of the different HLA alleles is determined by the dominant pathogens of each geographic region in particular, and because these genes are highly polymorphic.

Breast cancer has exhibited an increase in incidence in recent years, it is the tumor second only to lung cancer as cause of death by cancer in females, and is the number one cause of death by cancer in women 15 – 54 years of age worldwide [31]. In Mexico, breast cancer is a very frequent tumor; thus, study of this disease and the factors that predispose its presentation is of prime importance for identification of at-risk groups, which translates into a more precise evaluation for each woman [5].

To date, few studies have been conducted to attempt to determine the association and impact that these represent in the risk of presenting breast cancer and the different HLA, especially HLA class II, and some studies lack sufficient power due to a reduced number of studied cases [33].

In 2005 Lavado et al. [34], compared 132 women with breast cancer and 382 healthy controls in the Spanish region of Málaga. They performed HLA-A,-B, -Cw, -DR, and -DQ typification. The most important differences were found in the HLA-B locus, where the HLA-B7 allele was present with greater frequency in the group of sick patients than in the control group (*p *= 0.0019; 95% CI, 1.337 – 3.409; Relative risk [RR], 2.135), explaining that in this geographical zone an environmental agent can be found (whether viral or bacterial) that can be associated with breast cancer. Our study reveals a significantly increased frequency of HLA-B*1501 in cancer patients in comparison to healthy controls (OR = 3.714; CI95%, 1.187–11.619, *p *= 0.031) but not in other HLA-B alleles.

Gopalkrishnan et al. [36], in a group of women from India, evaluated low- or intermediate-resolution gene expression of HLA-A,-B, and -C, finding the following two alleles as candidates for markers associated in risk modulation for breast cancer in Eastern Indian women: Alleles HLA-B*40 and -B*08, the first as a factor for early development of the disease, presenting in 16% of cases vs. 9.0% of controls (OR, 2.2; 95% CI, 1.15 – 4.34; *p *= 0.02), and the second, found to be a protector. These protective or high risk alleles even though were frequent in our population (HLA-B*40 g.f.= 0.080 and -B*08 g.f. = 0.046) associations were not statistically significant neither for risk not for protection to the development of the neoplasm.

We found HLA-DQB1*0302 to be protective as well as HLA-DQB1*0301 but not associated with age, which is contrary to what was reported by Chaudhuri et al. [13] in 2000, where he reports two important negative associations for the development of breast cancer at an early age, both of HLA class II: DRB*11, which was found expressed in 35 controls and only in six cases (*p *< 0.0001). These results reflect, at least in the patient group, that inheritance of the alleles of these genes (DQB*03032 and DRB1*11) represent alleles resistant to the presentation of early-age breast cancer.

Positive association of specific HLA class II alleles in any malignant-tumor type reflects the specific role of these molecules in the promotion of chronic inflammation. HLA expression suggests that immune-system evasion of certain cellular populations could be responsible for promoting survival of the neoplasm, thus rendering it necessary to continue evaluating these markers in different populations and to include greater numbers of patients to confirm the different associations and risks between alleles and haplotypes and to determine whether there are others that could be catalogued as risk factors for development of the neoplasm, and at a determined moment whether the fact that some allele, alleles, or haplotypes are found expressed consistently in some group of individuals affords the power to utilize HLA class II typifications as prognostic factors, at the present moment few authors had performed characterization of HLA in latin population, we could say this is the first attempt to characterize a Mexican mestizo population in order to try to find associations between HLA and breast cancer.

## Conclusion

The results obtained by our group demonstrate the role of genetics in the multifactorial pathophysiology of breast malignant neoplasms. It also reveals the role of the MHC genes in the pathophysiology, suggesting that in the development of breast cancer exists a disorder of immune regulation.

Nevertheless, this triggering factor (MHC genes) seems to be restricted to certain ethnic groups as well as certain geographical regions since these relevant MHC alleles are highly diverse and confirms the relevance of HLA-DR alleles in the genetic susceptibility to develop this specific type of malignant disease.

## Abbreviations

HLA: human leukocyte antigen; MHC: Major histocompatibility; ACS: American cancer society; PCR: Polymerase chain reaction;

## Competing interests

The authors declare that they have no competing interests.

## Authors' contributions

DCL: Study design, patient recruitment, article writing. DPM: Data collection, article writing. VV: Patient recruitment, data collection. AGC: Molecular analysis. AMB: Statistic evaluation and data analysis. VAA: Sample analysis. ALT: Molecular analysis. GVA: Sample analysis. RB: Sample analysis. NY: Genetic analysis. EJY: Study design, data analysis. JG: Study design, manuscript evaluation, data analysis. All authors read and approved the final manuscript.

## Pre-publication history

The pre-publication history for this paper can be accessed here:

http://www.biomedcentral.com/1471-2407/9/48/prepub
